# Investigating the Dimensional Accuracy of the Cavity Produced by ABS P400 Polymer-Based Novel EDM Electrode

**DOI:** 10.3390/polym13234109

**Published:** 2021-11-25

**Authors:** Azhar Equbal, Asif Equbal, Zahid A. Khan, Irfan Anjum Badruddin, Mohamed Bashir Ali Bashir, Hussein Alrobei

**Affiliations:** 1 Department of Mechanical Engineering, Faculty of Engineering and Technology, Jamia Millia Islamia, New Delhi 110025, India; azhr_eqbl06@yahoo.co.in (A.E.); zakhanusm@yahoo.com (Z.A.K.); 2 Department of Mechanical Engineering, Cambridge Institute of Technology, Ranchi 835213, India; equbal.asif@yahoo.com; 3 Mechanical Engineering Department, College of Engineering, King Khalid University, Abha 61413, Saudi Arabia; 4 Department of Mechanical Engineering, College of Engineering, Jouf University, Sakaka 42421, Saudi Arabia; mbashir@ju.edu.sa; 5 Department of Mechanical Engineering, College of Engineering, Prince Sattam bin Abdullaziz University, Al-Kharj 16273, Saudi Arabia; h.alrobei@psau.edu.sa

**Keywords:** ABS P400 Polymer, fused deposition modeling, EDM, electrodes, metallization, dimensional accuracy

## Abstract

In the present study, cylindrical ABS P400 polymer parts (diameter 6.5 mm) to be used as die-sinking EDM (electric discharge machining) novel electrodes were fabricated using a fused deposition modeling (FDM) process. To meet the conductivity requirement in EDM, ABS parts were metallized using an innovative method that comprised putting aluminum–charcoal (Al–C) on them followed by their copper electroplating. Real-time EDM of the mild steel workpiece was performed using novel electrodes, and machining performance of the electrodes, measured in terms of dimensional accuracy, i.e., change in diameter (Δ*D*) and change in depth (Δ*H*) of the cavity, under varying levels of three EDM factors, i.e., current (*I*), pulse on time (*T_on_*), and pulse off time (*T_off_*), was investigated. Machining results were analyzed using analysis of variance (ANOVA), perturbation graphs, and 3D surface plots. The optimal setting of the EDM parameters for minimizing Δ*D* and Δ*H* was determined using the desirability function approach. The suitability of the novel electrodes for EDM was ascertained by comparing their machining results with those of solid copper (SC) electrodes and electrodes fabricated by FDM and metallized using the electro-deposition method (FDM-EM), already reported in the literature, under similar machining conditions. From the results, it was found that Δ*D* and Δ*H* were less when EDM was performed using novel electrodes.

## 1. Introduction

Electrical discharge machining (EDM) is a precision machining process in which repetitive controlled sparks are used to remove material from the conductive workpiece materials [1]. Sparks are produced between the tool (electrode) and the workpiece, which is completely submerged under an insulated working medium referred to as dielectrics [2,3,4]. When power is supplied, the dielectric strength breaks down at a particular voltage, and a plasma channel is established between the electrode and the workpiece, which helps in the formation of concentrated sparks. EDM is mainly employed for the machining of hard and brittle materials such as steel, titanium, tungsten, super-alloys, and ceramics [5,6,7]. Application areas of EDM include electronic industries, semi-conductor manufacturing industries, and mold and die industries [8,9]. In the mold and die industries, 25–40% of the total cost is incurred in the EDM of the workpiece, and 50% of EDM cost is spent in the design and production of the electrode, respectively [10]. The complexity of the cavity required to be machined further increases the cost of electrode production. Additive manufacturing (AM) comprises of processes that mainly use plastic-based polymers for the manufacturing of complex products, and recently, it has opened a new gateway for use of these products in novel applications such as mold and die making, electronics [10], medical uses [11,12,13], jewelry [14], and aerospace and automotive industries [15]. AM technology can be used to produce plastic-based low-cost complex-shaped EDM electrodes. Plastics are polymers that are made from repeating chains of small molecules known as monomers [11,12]. They are light in weight and possess characteristics such as water-resistance, corrosion resistance, and plasticity, etc. [10]. They are used in various applications ranging from household items such as buckets and bottles to engineering applications in automobiles, electronics, and textiles. They have become a very important part of modern society and have touched almost every aspect of it. However, the use of polymer in modern machining is rarely explored. To fill this gap to some extent, the authors of this paper have explored suitability of ABS P400 polymer for production of EDM electrodes. In the present study, cylindrical ABS P400 parts (diameter = 6.5 mm) were produced using the widely used fused deposition modeling (FDM) technique of AM. To meet the EDM requirement, the literature suggests well-established metallization methods for ABS parts [16,17,18,19]. However, in the present research, a novel route for the metallization of the ABS parts was adopted. Metallization was performed in the following two stages: (i) in the first stage, Al-C paste was provided on the ABS parts to induce conductivity, and (ii) in the second stage, copper electroplating of the Al-C coated parts was performed. Using the novel electrodes, real-time EDM of the mild steel workpiece was performed to produce cavities and machining performances of the electrode were measured.

Dimensional accuracy (*DA*) of the machined components is an important performance measure that determines the machining accuracy. The *DA* of a machined cavity is measured in terms of deviation in its diameter (Δ*D*) and depth (Δ*H*). As FDM has its own limitation in terms of dimensions of the fabricated parts [10,20], the *DA* of the cavity produced using an FDM-fabricated ABS-based EDM electrode is an important subject of investigation. Moreover, only a few researchers have investigated the dimensional accuracy of the machined cavity produced by FDM-fabricated EDM electrodes and compared it with that obtained using solid copper electrodes [10]. Present research is thus aimed at investigating the *DA* of the machined cavity produced by the novel ABS-based EDM electrodes. To validate the machining results, the *DA* of the machined cavity achieved using the novel electrodes is compared with that of solid copper (SC) electrodes and electrodes fabricated by FDM and metallized using electro-deposition method (FDM-EM) already reported in literature under similar machining conditions. The optimal setting of the EDM parameters for minimizing both Δ*D* and Δ*H* is also determined using the desirability function approach, which is an important optimization method widely used in machining, casting, forging, and other medical applications [21].

## 2. Methodology

**Figure 1:**
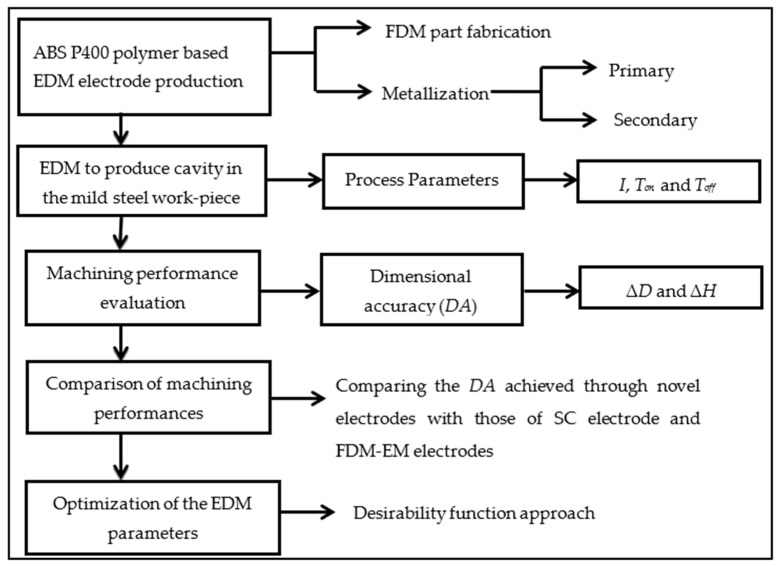
A schematic diagram of the adopted methodology.

The methodology used to accomplish the research objective comprised several stages, which included electrode production, machining of the cavity, machining performance evaluation, comparison of machining performances, and optimization of EDM parameters. A schematic diagram, showing these stages, is presented in Figure 1.

### 2.1. Electrode Production

**Figure 2:**
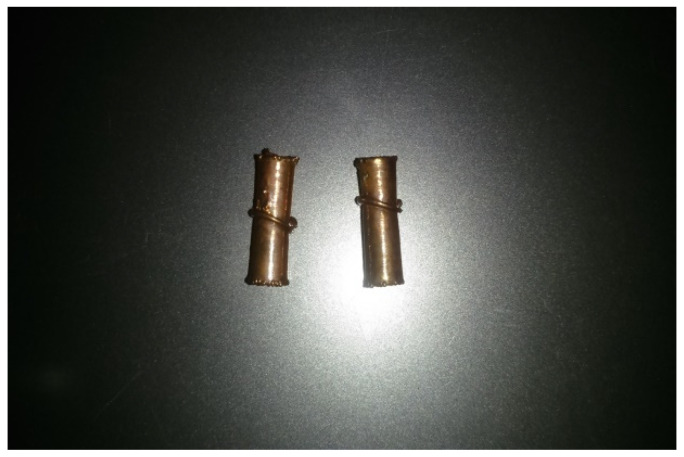
Novel ABS P400 electrode.

The electrode was produced in two stages. Initially, cylindrical parts (length l = 50 mm) were fabricated by an FDM (fused deposition modeling) machine (FDM Vantage SE Make, Stratasys Unit, Pune, India) by depositing ABS P400 (Stratasys Unit, Pune, India) in a layer-wise manner. Parts were fabricated at the optimal setting of the FDM parameters, i.e., raster angle = 0, air gap = −0.004 mm, and raster thickness = 0.5064 mm, to achieve better dimensional accuracy, minimum surface roughness, and high compressive strength [10]. Since high compressive stress is developed at the inner core of the electrode during EDM and FDM parts served as the inner core of electrode developed in the present research, the compressive strengths of FDM parts were measured [22]. Metallization of the FDM fabricated parts was performed in the second stage. Metallization itself was performed in two stages. For primary metallization, aluminum powder, activated charcoal powder, enamel, and distilled water were mixed in a weight ratio of 40:3:36:21, and a viscous paste was prepared by vigorously mixing them in a magnetic stirrer [10]. The prepared Al-C paste was then manually applied on the FDM parts using a soft brush, and the pasted parts were dried completely. Dried parts were then scoured with sandpaper to allow the maximum exposure of aluminum over the entire surface of the parts. The scoured parts were then subjected to copper electroplating (using standard electroplating apparatus), having a bath concentration comprising 200 g/L of Copper sulphate (CuSO_4_), 60 mL/L of Sulfuric acid (H_2_SO_4_), and 120 mL/L of Hydrochloric acid (HCl). Trial electroplating at higher current densities, i.e., ≥4 A/dm^2^, resulted in the burning of the metallized layer and thus the current density of 3 A/dm^2^ was selected for electroplating of the Al-C pasted parts. In accordance with Equation (1) [10], electroplating was performed at 3 A/dm^2^ for 5 h to achieve the coating thickness of 220 µm.

(1) $$T^{2}=-5.3571t^{2}+101.09t-154.94$$

where, *T* is the coating thickness in mm, and *t* is the deposition time in hrs. The detailed methodology for selection of optimal parameters is available in author’s previous work [9,10]. In the electroplating stage, it was observed that the thickness of the coating at different locations of the electrode was slightly different from 220 µm. However, the average thickness was 220 µm, which was measured with the help of Vernier calipers by averaging the thickness taken at five different locations. After metallization, the diameter of the fabricated electrodes was measured in the range of $6.5_{-0.005}^{+0.010}$ mm. The fabricated electrode is shown in Figure 2. Due to the limitations of the FDM process and the metallization procedure, the exact dimension of the electrode could not be achieved.

### 2.2. Experimental Design, EDM, and Performance Measures

EDM with the novel electrodes was performed in accordance with the experimental matrix (Table 1) obtained from response surface methodology (RSM)-based face centered central composite design (FCCCD). The experimental matrix used three factors, each at three different levels, and fewer center runs used by other CCD designs. EDM parameters and their levels were chosen from the literature, and the same were used for evaluating machining performances of SC and FDM-EM electrodes [10] and are shown in Table 2.

EDM was performed to create cavities of diameter 6.5 mm and depth 2 mm according to the RSM-based FCCCD design, as shown in Table 1. Mild steel was selected as the workpiece material, and EDM was performed using the EDM machine (Vidyunt MMT ZNC, Pune, India). After machining, the data for dimensional accuracy, in terms of deviation in diameter (Δ*D*) and deviation in depth (Δ*H*) of the machined cavities, were collected using a Vernier caliper (Make—Aerospace digital caliper; least count = 0.01 mm). Δ*D* was measured by subtracting 6.5 mm from the obtained machined diameter, and Δ*H* was computed by subtracting 2 mm from the obtained machined depth. For each machined cavity, five values of Δ*D* and Δ*H* were measured at five different locations and then their average value was obtained, which was considered for analysis.

### 2.3. Analysis of Results, Comparisons, and Optimization

Analysis of variance (ANOVA) was used for analyzing the experimental results. ANOVA is a decision-making tool that describes a polynomial relation between input constraints and output responses [10]. The determination of parameters’ and interactions’ significance is performed by calculation of the *p*-value. For a 5% significance level, terms or interactions having a *p*-value ≤ 0.05 were considered as significant and vice-versa. A normality plot was constructed for establishing the effectiveness of the developed model. Here, the *p*-value should be ≥ 0.05, and if the *p*-value ≤ 0.05, then it was inferred that data were not distributed normally.

**Figure 3:**
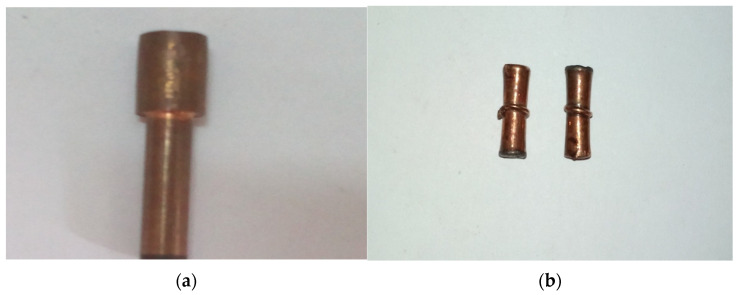
(**a**) SC electrode, (**b**) FDM-EM electrode.

To establish their suitability in EDM application, machining results of the novel electrode were compared with those of the solid copper (SC) (Figure 3a) electrode and electrodes fabricated by FDM and metallized using electro-deposition method (FDM-EM) (Figure 3b), already reported in the literature under similar machining conditions [10]. Finally, the desirability function approach was used to determine the optimal setting of the EDM parameters, which minimized both Δ*D* and Δ*H*.

## 3. Results and Discussions

**Figure 4:**
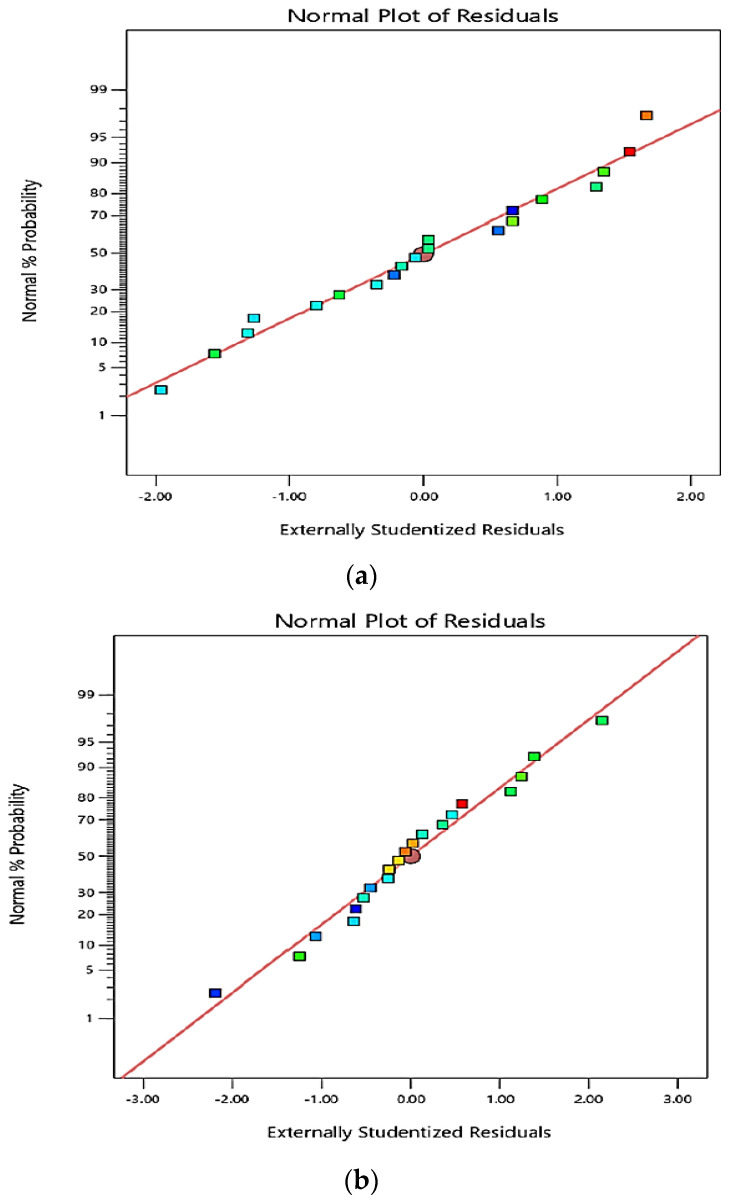
Normality plot for (**a**) Δ*D* and (**b**) Δ*H*.

Results for dimensional accuracy, i.e., Δ*D* and Δ*H*, are shown in Table 3. ANOVA results for Δ*D* and Δ*H* are presented in Table 4. Here, in the ANOVA table, *MS*, *SS*, and *DOF* signify mean square or variance, sum of square, and degree of freedom, respectively. *R*^2^ represents the coefficient of variance, and *LOF* symbolizes the lack of fit. The F value is the ratio of explained variance to unexplained variance. The percentage variation in the machining performance explained by the model was determined using the value of *R*^2^. Pure error reflects the variability of the observations within each treatment, and residual error means unexplained variance. ANOVA results presented in Table 4 reveal that *R^2^* is greater than 0.80 for both Δ*D* and Δ*H*, which implies that the models can explain more than 80% (83.9% for Δ*D* and 87.7% for Δ*H*) of variations in the machining performance. It is evident from Table 4 that, for Δ*D*, the EDM parameters *I* and *T_on_* are significant as the *p*-values for them are less than 0.05; however, for Δ*H*, only *I* is significant. Response surface equations for Δ*D* and Δ*H* are given in Equations (2) and (3). Figure 4a,b presents the normality plots for Δ*D* and Δ*H*, respectively. Figure 4a,b shows that errors are very close to the line and therefore the data is assumed to be normally distributed. Here, different colors represent the distribution of values in different regions of response surface after conversion. For example, red means the value that lies in the region of 90% or the response surface with a hotter extreme region. Since the value of *R^2^* for Δ*D* and Δ*H* is 0.839 and 0.877, respectively, it appears that the models developed for Δ*D* and Δ*H* given in Equations (2) and (3) are in good agreement for predicting the response values studied.

(2) $$\begin{array}{l} \Delta D=0.083236+0.027200\times I+0.044000\times T_{on}+0.011000\times T_{off}+0.012500\times I\times T_{on}\\ -0.0050\times I\times T_{off}+0.005000\times T_{on}\times T_{off}+0.022909\times I^{2}-0.003091\times T_{on}{}^{2}-0.008091\times T_{off}{}^{2} \end{array}$$

(3) $$\begin{array}{l} \Delta H=-0.073273+0.055000\times I-0.012000\times T_{on}+0.009000\times T_{off}+0.022500\times I\times T_{on}\\ -0.0100000\times I\times T_{off}-0.005000\times T_{on}\times T_{off}+0.043182\times I^{2}+0.018182\times T_{on}{}^{2}-0.006818\times T_{off}{}^{2} \end{array}$$

**Figure 5:**
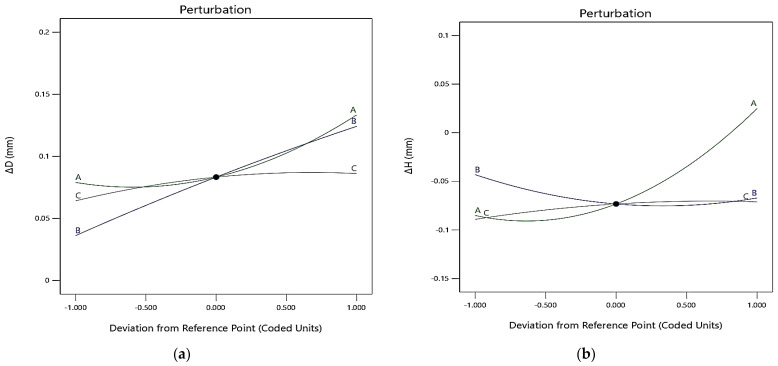
Perturbation graph for (**a**) Δ*D* and (**b**) Δ*H*.

3D surface plots and perturbation graphs were used for analyzing the influence of EDM parameters on the machining performances. Table 4 shows that for Δ*D*, the parameters *I* and *T_on_* are significant, but for Δ*H*, only current (*I*) is significant. When it comes to interactions, it is found that only *I* × *T_on_* is significant for Δ*H*, but for Δ*D*, none of the interaction terms are significant. However, to explain the variations in Δ*D* and Δ*H* with change in input parameters, 3D surface plots for all the interactions are explained. Figure 5a,b presents the perturbation graph for Δ*D* and Δ*H*. Here A, B, and C represent *I*, *T_on_*, and *T_off_*, respectively. It is evident from Figure 5a that, with the increase in *I*, Δ*D* is not much affected in the beginning, but once the value of *I* is sufficiently increased, Δ*D* continues to increase. Further, with the increase in *T_on_*, Δ*D* increases uniformly; however, with the increase in *T_off_*, Δ*D* increases in the beginning but, as the machining progresses, Δ*D* becomes almost constant.

**Figure 6:**
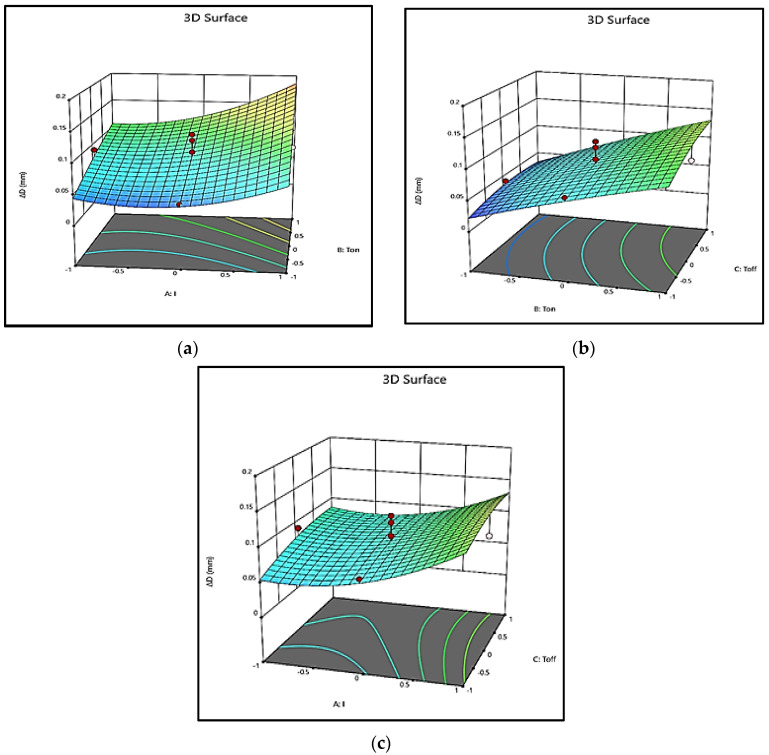
3D Surface plot for Δ*D* (**a**) *I* × *T_on_*; (**b**) *T_on_* × *T_off_*; (**c**) *I* × *T_off_*.

The same conclusions can be drawn from 3D surface plots presented in Figure 6. Figure 6a shows that with the increase in *I*, the energy of the spark increases, but at a low value of *I*, the intensity of the spark is low, and hence, Δ*D* is not much affected. Once *I* attain a higher value, the intensity of the spark is much more and causes an increase in Δ*D*. With the increase in *T_on_*, the diameter of plasma channel spreads, and hence, the heat transfer to the tool reduces and the heat transfer to the workpiece increases [23], which may lead to a uniform increase in Δ*D* (Figure 6a,b). With the rise in *T_on_*, the phenomenon is continued, and a further increase in Δ*D* is observed. The increase in Δ*D* is also found with a rise in *T_off_* (Figure 6b,c). However, the increase in Δ*D* is much more noticeable in the initial stage of machining when the *T_off_* is low. At higher *T_off_*, the interval between continuous sparks increases, and the dielectric obtains a sufficient time to re-establish its strength [24]. As some energy is utilized in overcoming the regained dielectric strength, the available energy at high *T_off_* causes an insignificant increase in Δ*D*.

**Figure 7:**
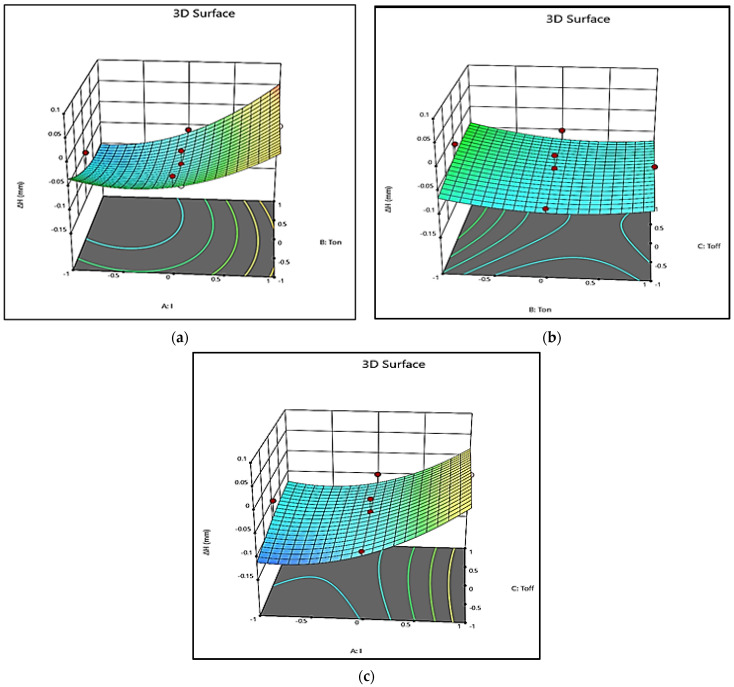
3D Surface plot for Δ*H* (**a**) *I* × *T_on_*; (**b**) *T_on_* × *T_off_*; (**c**) *I* × *T_off_*.

Figure 5b shows the perturbation graph for Δ*H*. Here also, A, B, and C represent *I*, *T_on_*, and *T_off_*, respectively. It is evident from Figure 5b that Δ*H* increases with an increase in *I*. The increase in Δ*H* is, however, more when the value of current is high. The increase in *T_on_* causes a decrease in Δ*H*. However, an increase in *T_off_* leads to a marginal increase in Δ*H*. The same conclusions can be drawn from 3D surface plots presented in Figure 7. With the increase in *I*, Δ*H* increases marginally in the beginning, as the spark is not very intense. At a higher value of *I*, the intensity of the spark is high, which may lead to a rapid increase in Δ*H* (Figure 7a,c).

Figure 7b shows that a higher *T_on_* spread of the plasma channel results in an increase in Δ*D*; on the other hand, Δ*H* decreases because the spread of spark energy decreases its strength, which causes difficulty in deeper machining. Figure 7b,c also depicts that with an increase in *T_off_*, the increase in Δ*H* is marginal, since at higher *T_off_*, the frequency of sparks decreases, and a significant portion of the spark energy is utilized in overcoming the regained dielectric strength and hence energy to create a deeper slot is lacking, which leads to a marginal increase in Δ*H*. In Figure 6 and Figure 7, color bars depict the region of intensities in the response surface plots.

## 4. Optimization

To determine the optimal setting of the EDM parameters (*I*, *T_on_* and *T_off_*) at which Δ*D* and Δ*H* are minimum, the desirability function approach was used [21]. The desirability approach is a local optimization technique that is used to determine the optimum setting within the specified range. It determines the optimum condition that yields the most desirable input. All the responses are scaled to a uniform range [0, 1] using the suitable conditions (namely larger-the-better, smaller-the-better, and nominal the better) and standard equations [25]. After converting the responses to the values 0 and 1, individual desirability values are calculated. After calculating the individual desirability, overall desirability or composite desirability is calculated by using Equation (4).

(4) $$D=\;(d_{1}\times d_{2}\times d_{3}\times \ldots d_{n})\;={\left(\overset{n}{\underset{i=1}{\Pi }}d_{i}\right)}^{1/n}$$

where *D* is composite desirability, *d*_1_, *d*_2_, *…*, *d_n_* are the maximum desirable values for different response, and *n* is the number of responses. The maximum desirability value then was chosen, and the factor setting corresponding to maximum desirability is then selected as the optimum factor setting.

**Figure 8:**
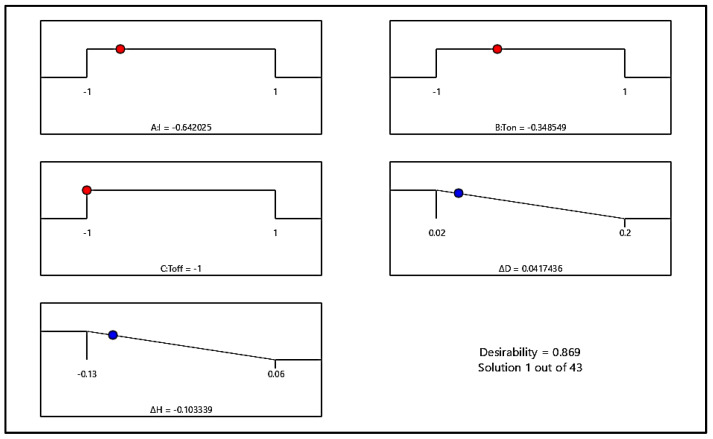
Composite desirability for Δ*D* and Δ*H* at optimal setting of *I*, *T_on_*, and *T_off_*.

In the present study, both responses Δ*D* and Δ*H* were to be minimized and hence the goal for both the responses was set at minimization. Equal importance was assigned to both the output responses. The obtained optimum setting of EDM parameters and optimal results for Δ*D* and Δ*H* are shown in Figure 8. It was found that the optimal parameter setting of the EDM parameters in coded form was *I* = −0.642025, *T_on_* = −0.348549, and *T_off_* = −1. The equivalent actual values corresponding to these coded values were obtained from Equation (5) [10], and they were *I* = 4.07 A, *T_on_* = 148.68 µs, and *T_off_* = 90 µs. The optimum values of Δ*D* and Δ*H* were 0.0417436 and −0.103339 mm, respectively.

(5) $$\begin{array}{l} \xi_{ij}=\left(\frac{x_{ij}-\bar{x_{i}}}{\Delta x_{i}}\right)\times 2\\ \bar{x_{i}}=\frac{\sum_{j=1}^{2}x_{ij}}{2},\;\mathrm{and}\;\Delta x_{i}=x_{i2}-x_{i1} \end{array}$$

where, 1 ≤ *i* ≤ *k*; 1 ≤ *j* ≤ 2 and $\xi_{ij}$, *x_ij_* are coded and actual value of the *j*th level of *i*th factor, $\bar{x_{i}}$ is mean of values for factor *i*.

## 5. Comparison of Machining Performances

Machining performances of the novel electrode were compared with those of the solid copper (SC) electrode and electrodes fabricated by FDM and metallized using the electro-deposition method (FDM-EM) under similar machining conditions already reported in the literature [10] for assessing its efficacy in EDM application. Comparison of Δ*D* and Δ*H* are presented in Table 5. During EDM, there is continuous removal of material from the workpiece and simultaneously there is micro-wear in the electrode used for machining due to which there is continuous variation in the gap between the electrode and workpiece, and therefore, it is difficult to maintain a uniform gap during the process. Consequently, it is not possible to obtain the desired diameter of machined cavity using any of the electrodes, and deviation in the diameter is bound to be there, as shown in Table 5. Table 5 also reveals that for the entire machine settings, Δ*D* achieved using novel electrodes is less when compared with Δ*D* obtained using SC electrodes, which signifies that the machining accuracy of novel electrodes is better than that of SC electrodes. It is also evident from Table 5 that the pattern of Δ*D* achieved using FDM-EM electrodes is not uniform, and machined cavities are either undersized (negative value of Δ*D*) or oversized. Further, Δ*D* produced using FDM-EM electrodes is comparable to the results of novel electrodes at machine settings, which produced oversized cavities. In addition, the undersize cavities produced by FDM-EM electrodes require further machining to obtain the required dimensions, which will increase the machining cost. Moreover, precise machining to bring the cavities to exact diameter is also a challenging task. Overall analysis suggests that Δ*D* achieved through a novel electrode is better when compared with Δ*D* produced by SC electrodes and FDM-EM electrodes. The same conclusion is also proposed by the root sum mean of square calculated for the entire used electrode. The root sum mean of the square for Δ*D* is less when a novel electrode is used for EDM when compared with the SC electrode and FDM-EM electrodes.

Comparison of Δ*H* obtained using different electrodes is also presented in Table 5, which manifests that Δ*H* achieved using novel electrodes and FDM-EM electrodes is either less (negative value of Δ*H*) or more (positive value of Δ*H*) than the required machined depth. Further, Table 5 also shows that, whether depth of the cavities is less or more than the desired depth, least variation in Δ*H* is observed when machining is performed using novel electrodes, which suggests that machining with a novel electrode is more accurate than the FDM-EM electrode. For both novel electrodes as well as FDM-EM electrodes, non-uniform deposition of copper at corners of primary metallized parts is responsible for inexact depth produced during EDM. The depth of the cavities produced with SC electrodes is always more than the required depth, as it is a solid electrode, and there is no problem of copper deposition at corners like in novel electrodes and FDM-EM electrodes. Here also, the root sum mean of square for Δ*H* is less when the novel electrode is used for EDM when compared with the SC electrode and FDM-EM electrodes.

## 6. Conclusions

Novel ABS P400 polymer-based EDM electrodes were produced first by fabricating cylindrical ABS parts using the FDM technique and then metalizing them by providing aluminum–charcoal (Al–C) paste on them followed by copper electroplating. An investigative study was carried out on the dimensional accuracy of the EDM machined cavities created using novel electrodes. Three critical EDM parameters, i.e., current (*I*), pulse on time (*T_on_*) and pulse off time (*T_off_*), under varying levels were studied to determine their effect on deviations in diameter (Δ*D*) and depth (Δ*H*) of the machined cavity. Machining results were analyzed using analysis of variance (ANOVA), perturbation graphs, and 3D surface plots. The optimal setting of the EDM parameters for minimizing Δ*D* and Δ*H* was determined using the desirability function approach. Effectiveness of the novel electrode was established by comparing its machining results with those of solid copper (SC) electrodes and electrodes fabricated by FDM and metallized using the electro-deposition method (FDM-EM) already reported in literature under similar machining conditions. The following important conclusions are drawn:

(1). Δ*D* achieved using novel electrodes is less when compared with Δ*D* obtained using SC electrodes.
(2). Δ*D* produced by FDM-EM electrodes is comparable to Δ*D* achieved using novel electrodes at machine settings, which produces oversize cavities.
(3). Undersize cavities produced using FDM-EM electrodes require further machining to achieve the desired dimension, which increases the machining cost.
(4). Machining depth obtained with novel electrodes and FDM-EM electrodes is either less or more than the desired depth. However, the least variation in Δ*H* is observed when machining is performed using novel electrodes.
(5). For both novel electrodes and FDM-EM electrodes, non-uniform deposition of copper at corners of primary metallized parts is responsible for the inexact depth produced during EDM.
(6). The depth of cavities produced with SC electrodes is always more than the required depth, as it is a solid electrode, and there is no problem of copper deposition at corners like in novel electrodes and FDM-EM electrodes.
(7). Δ*D* is significantly affected by *I* and *T_on_*, whereas *I* is the more dominating factor for Δ*H*.
(8). From the result obtained, it is inferred that better dimensional accuracy is provided by novel electrodes when real-time machining was performed. It is also suggested that novel electrodes are recommended when finish machining is required using EDM, as the variations in Δ*D* and Δ*H* are minimum. In the real practice, the dimensional accuracy produced by the novel electrode is affected by inherent dimensional inaccuracy in FDM-fabricated parts as well as non-uniform deposition at the corners of the electrode due to continuous variation in current density.
(9). Desirability-based optimization shows that for minimum Δ*D* and minimum Δ*H*, the optimal setting of the EDM parameters is *I* = 4.07 A, *T_on_* = 148.68 µs, and *T_off_* = 90 µs in coded form, and the values of Δ*D* and Δ*H* at the optimized setting are 0.0417436 and −0.103339 mm respectively.

## Figures and Tables

**Table 1:** Experimental matrix obtained from RSM-based FCCCD design [10].

Exp. No.	Factors (Coded)		
	*I*	*T_on_*	*T_off_*
1	−1	−1	−1
2	1	−1	−1
3	−1	1	−1
4	1	1	−1
5	−1	−1	1
6	1	−1	1
7	−1	1	1
8	1	1	1
9	−1	0	0
10	1	0	0
11	0	−1	0
12	0	1	0
13	0	0	−1
14	0	0	1
15	0	0	0
16	0	0	0
17	0	0	0
18	0	0	0
19	0	0	0
20	0	0	0

**Table 2:** EDM parameters and their levels [10].

EDM Parameter	Symbol	Levels			Unit
		1	2	3	
		Low Level (−1)	Center Level (0)	High Level (+1)	
Current	*I*	3	6	9	A
Pulse on time	*T_on_*	90	180	270	µs
Pulse on time	*T_off_*	90	120	150	µs

**Table 3:** Experimental results for machining performances.

Exp. No.	Δ*D* (mm)	Δ*H* (mm)
1	0.020	−0.07
2	0.060	0.02
3	0.070	−0.13
4	0.180	0.06
5	0.040	−0.03
6	0.080	0.03
7	0.130	−0.10
8	0.200	0.04
9	0.090	−0.05
10	0.102	0.02
11	0.040	−0.02
12	0.100	−0.06
13	0.070	−0.08
14	0.060	−0.05
15	0.090	−0.04
16	0.060	−0.09
17	0.110	−0.10
18	0.070	−0.07
19	0.120	−0.08
20	0.090	−0.12

**Table 4:** ANOVA results for Δ*D* and Δ*H*.

Source	*DOF*	Δ*D*				Δ*H*			
		*SS*	*MS*	*F*	*p*-Value	*SS*	*MS*	*F*	*p*-Value
*I*	1	0.0074	0.0074	12.34	0.006	0.0302	0.0302	42.48	0.000
*T_on_*	1	0.0194	0.0194	32.28	0.000	0.0014	0.0014	2.02	0.186
*T_off_*	1	0.0012	0.0012	2.02	0.186	0.0008	0.0008	1.14	0.3113
*I* × *T_on_*	1	0.0013	0.0013	2.08	0.179	0.0041	0.0041	5.69	0.0383
*I* × *T_off_*	1	0.0002	0.0002	0.33	0.576	0.0008	0.0008	1.12	0.3141
*T_on_* × *T_off_*	1	0.0002	0.0002	0.33	0.576	0.0002	0.0002	0.2809	0.6077
*I* × *I*	1	0.0014	0.0014	2.41	0.152	0.0051	0.0051	7.20	0.0230
*T_on_* × *T_on_*	1	0.0000	0.0000	0.04	0.838	0.0009	0.0009	1.28	0.2849
*T_off_* × *T_off_*	1	0.0002	0.0002	0.30	0.596	0.0001	0.0001	0.1795	0.6807
Residual error	10	0.0060	0.0006	--	--	0.0071	0.0007	--	--
*LOF*	5	0.0034	0.0007	1.31	0.388	0.0034	0.0007	0.9074	0.5412
Pure Error	5	0.0026	0.0005	--	--	0.0037	0.0007	--	--
Total	19	0.0372	--	--	--	0.0581	--	--	--
		*R*^2^ = 0.839				*R*^2^ = 0.877			

**Table 5:** Comparison of Δ*D* and Δ*H*.

Exp. No.	Factors (Coded)			Δ*D* (mm)			Δ*H* (mm)		
	*I*	*T_on_*	*T_off_*	Novel Electrode	SC Electrode [10]	FDM-EM Electrode [10]	Novel Electrode	SC Electrode [10]	FDM-EM Electrode [10]
1	−1	−1	−1	0.020	0.21	−0.06	−0.07	0.07	−0.12
2	1	−1	−1	0.060	0.13	−0.02	0.02	0.05	0.04
3	−1	1	−1	0.070	0.20	0.05	−0.13	0.04	−0.20
4	1	1	−1	0.180	0.26	0.29	0.06	0.11	−0.04
5	−1	−1	1	0.040	0.22	0.03	−0.03	0.03	−0.01
6	1	−1	1	0.080	0.20	−0.10	0.03	0.04	0.02
7	−1	1	1	0.130	0.15	0.18	−0.10	0.03	0.09
8	1	1	1	0.200	0.32	0.31	0.04	0.11	−0.02
9	−1	0	0	0.090	0.22	−0.15	−0.05	0.04	−0.09
10	1	0	0	0.102	0.24	−0.02	0.02	0.11	0.03
11	0	−1	0	0.040	0.25	−0.11	−0.02	0.04	−0.01
12	0	1	0	0.100	0.30	−0.04	−0.06	0.07	−0.16
13	0	0	−1	0.070	0.27	0.11	−0.08	0.09	−0.02
14	0	0	1	0.060	0.29	0.05	−0.05	0.09	0.00
15	0	0	0	0.090	0.31	−0.15	−0.04	0.07	−0.02
16	0	0	0	0.060	0.30	−0.10	−0.09	0.11	−0.14
17	0	0	0	0.110	0.32	−0.06	−0.10	0.15	−0.16
18	0	0	0	0.070	0.30	−0.08	−0.07	0.11	−0.14
19	0	0	0	0.120	0.32	−0.08	−0.08	0.13	−0.14
20	0	0	0	0.090	0.31	−0.09	−0.12	0.10	−0.13
Root sum mean of square				0.099	0.262	0.130	0.071	0087	0.101

## Data Availability

The data available in manuscript.
